# Hemodynamic instability during percutaneous ablation of extra-adrenal metastases of pheochromocytoma and paragangliomas: a case series

**DOI:** 10.1186/s12871-018-0626-1

**Published:** 2018-11-06

**Authors:** Atousa Deljou, Jacob D. Kohlenberg, Toby N. Weingarten, Irina Bancos, William F. Young Jr, Darrell R. Schroeder, David P. Martin, Juraj Sprung

**Affiliations:** 1 Department of Anesthesiology and Perioperative Medicine, Mayo Clinic College of Medicine and Science, 200 First Street SW, Rochester, MN 55905 USA; 2 Department of Internal Medicine, Mayo Clinic, Rochester, MN USA; 3 Division of Endocrinology, Diabetes, Metabolism, and Nutrition, Mayo Clinic, Rochester, MN USA; 4 Division of Biomedical Statistics and Informatics, Mayo Clinic, Rochester, MN USA

**Keywords:** Ablation, Hypertension, Metastatic paragangliomas, Metastatic pheochromocytomas, Radiology suite

## Abstract

**Background:**

Surgical manipulation of pheochromocytomas and paragangliomas (PPGLs) may induce large hemodynamic oscillations due to catecholamine release. Little is known regarding hemodynamic instability during percutaneous ablation of PPGLs. We examined intraprocedural hemodynamic variability and postoperative complications related to percutaneous ablation of extra-adrenal metastases of PPGL.

**Methods:**

From institutional PPGL registry we identified patients undergoing ablation of extra-adrenal PPGL metastases from January 1, 2000, through December 31, 2016. We reviewed medical records for clinical characteristics and hospital outcomes. Tumors were categorized as *functional* or *nonfunctional* based on preprocedural fractionated catecholamine and metanephrine profiles.

**Results:**

Twenty-one patients (14 female [67%]) underwent 38 ablations. Twenty-four ablations were performed in patients with functional metastatic lesions, and 14 were in nonfunctional lesions. Intraprocedural use of potent vasodilators for hypertension was higher for patients with functional tumors (*P* = 0.02); use of vasopressors for hypotension was similar for functional and nonfunctional tumors (*P* = 0.74). Mean (±SD) intraprocedural blood pressure range (maximum–minimum blood pressure) during 38 procedures was greater for functional than nonfunctional tumors [systolic: 106 (±48) vs 64 (±30) mm Hg, *P* = 0.005; diastolic: 58 (±22) vs 35 (±14) mm Hg, *P* = 0.002; mean arterial: 84 (±43) vs 47 (±29) mm Hg, *P* = 0.007]. Complications included 5 unplanned intensive care unit admissions (3 for precautionary monitoring, 1 for recalcitrant hypotension, and 1 for hypertensive crisis), 1 case of postoperative bleeding, and 1 death.

**Conclusions:**

Substantial hemodynamic instability may develop during ablation of functional and nonfunctional PPGL metastases. When anesthesia is provided for ablation of metastatic PPGLs in radiology suites, preparation for hemodynamic management should match standards used for surgical resection.

## Background

Pheochromocytomas and paragangliomas (PPGLs) are neuroendocrine tumors. The annual incidence of PPGLs is 0.8 per 100,000 person-years [1], and they affect an estimated 500 to 1600 patients per year in the United States [2]. An estimated incidence of malignant PPGL in the United States in 2002 was 93 per 400 million persons [3]. Although primary PPGLs are usually resected surgically, metastatic lesions can be treated using percutaneous imaging-guided thermal ablation [4, 5]. This is considered an optimal approach for treatment of focal unresectable metastatic lesions in the liver, bone, and lungs [6, 7]. Two major modalities of ablation are hyperthermic (radiofrequency ablation) and hypothermic (cryoablation).

Surgical manipulation of PPGLs may induce hemodynamic oscillations due to catecholamine release from the tumor. Before the routine clinical introduction of preprocedural adrenergic blockade, resection of PPGL was associated with high morbidity and mortality [8]. Currently, it is a widely established practice for patients with PPGLs to undergo preprocedural pharmacologic treatment with α-adrenergic receptor blockers or calcium channel blockers [9–12]. This practice is designed to attenuate hypertensive crises during tumor manipulation. However, despite adrenergic receptor blockade, hemodynamic volatility is still frequently observed [13, 14]. We recently reported hemodynamic oscillations during open and laparoscopic operations of PPGL [15, 16]; however, we lack the knowledge regarding the extent of hemodynamic fluctuations during ablation of extra-adrenal PPGL metastases. In this study, we aim to describe intraprocedural hemodynamics for patients with nonfunctional and functional metastatic PPGL lesions, and their periprocedural outcomes.

## Methods

This study was approved by the Mayo Clinic Institutional Review Board in November 2014 (No. 13–004137), with the last modification approved in November 2017 (No. 14–008336). In compliance with Minnesota Statute 144.335, all patients during Mayo Clinic visits are asked to provide written informed research consent, and those who refuse are excluded from reviews (in general this represents less than 5% of patients). All patients included in the present study had research authorization on file. This study conformed to the requirements of the STROBE (Strengthening the Reporting of Observational Studies in Epidemiology) guidelines.

### Patient selection

For this historical cohort study, we searched the Mayo Clinic, Rochester, Minnesota, PPGL Registry maintained by the Division of Endocrinology, Diabetes, Metabolism, and Nutrition to identify adult patients who were treated for metastatic lesions in radiology suites using minimally invasive thermal ablation, from January 1, 2000, through December 31, 2016. This study partially overlaps with an earlier report from our institution that focuses on efficacy and safety of radiofrequency ablation and cryoablation therapy [17].

### Preprocedural adrenergic receptor blockade

For patients with PPGL, the aim of preoperative pharmacologic preparation is to blunt hemodynamic oscillations related to catecholamines released during procedural manipulation. Exceptions to the use of blockade are nonsecretory parasympathetic-derived skull base and neck PPGLs [18]. The details of our protocol used before surgical resection have been previously reported [13, 15, 16]. Our practice for preablation preparation of patients with metastatic PPGL lesions includes administering the same adrenergic blocking agents that are used for the surgical resection of a primary PPGL [19]. Briefly, treatment with phenoxybenzamine, a noncompetitive α_1,2_-adrenoceptor antagonist, is initiated 7 to 14 days before intervention. Alternative treatment is with selective α_1_-adrenergic blockers (eg, doxazosin). If the heart rate remains above 80 beats/min, a β-adrenergic receptor antagonist is added 2 to 5 days before surgery. The drugs are titrated to effect; if normotension is not achieved, a calcium channel blocker may be added. For patients with inadequate response to therapy (with low-normal blood pressures), or if a large release of catecholamines is anticipated, metyrosine is added to block the rate-limiting step of catecholamine synthesis.

### Data abstraction

For identified patients, medical, surgical, and anesthesia records were reviewed as previously described [20]. We abstracted data on demographics; preoperative pharmacologic preparation; preoperative blood pressures, major comorbid conditions (diabetes mellitus, cardiac disease, stroke or history of transient ischemic attack), and medications used for preoperative control of blood pressure and heart rate (α- and β-adrenergic receptor antagonists, calcium channel blockers, and metyrosine); location and functional status of the primary tumor and metastatic lesions; and type of procedure (cryoablation, radiofrequency ablation). Tumor status was designated as *functional*, from preprocedural plasma and 24-hour urine fractionated metanephrine levels, if the concentration of at least 1 of the following was higher than the reference range: urine total metanephrines (≥1300 mcg/24 h), urine metanephrine (≥400 mcg/24 h), urine normetanephrine (≥900 mcg/24 h), plasma-free metanephrine (≥0.5 nmol/L), or plasma normetanephrine (≥0.9 nmol/L).

From our electronic anesthesia database, we retrieved the maximum and minimum systolic, diastolic, and mean arterial blood pressures and heart rates for each procedure. Intraoperative blood pressure and heart rate variation were quantified using the range of the intraoperative values for a given patient (difference between maximum and minimum values recorded during the procedure). Intraoperative variables reviewed were any infusion of potent vasoactive drugs, intravenous fluids, and blood products. Perioperative complications (postoperative hemodynamic instability requiring treatment, unplanned intensive care unit [ICU] admission, bleeding, and death) were also abstracted. Only complications that occurred during index hospitalization are described in this report.

### Statistical analysis

Categorical data are presented as numbers (percentages), and continuous data are reported as mean (±SD) or median (interquartile range). Comparison of intraoperative hemodynamics between patients with functional and nonfunctional tumors was performed with the Fisher exact test or *t* test; 2-tailed *P* values 0.05 or less were considered significant. Because some patients underwent multiple ablations, analyses were performed including only the first ablation per patient and also including all ablations. For analyses performed using all ablations we assumed that all ablations are independent. Statistical analyses were performed with SAS software, version 9.4 (SAS Institute Inc).

## Results

### Patient characteristics

**Fig. 1:**
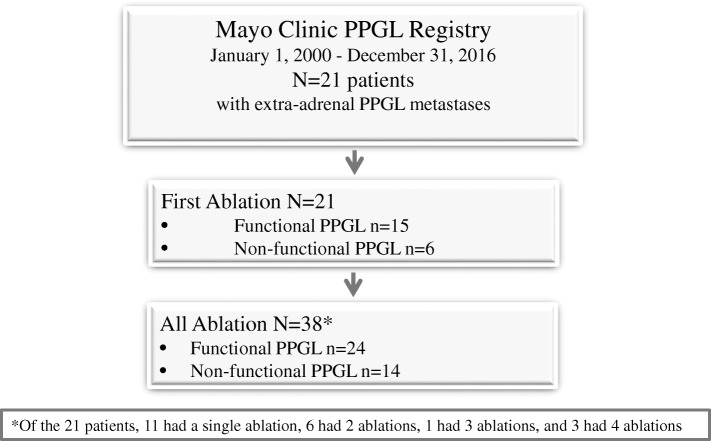
Summary of patients and ablation episodes of extraadrenal PPGL metastatic lesions included in the present report. PPGL = pheochromocytoma and paraganglioma

We identified 21 patients (14 female [67%]) with metastatic PPGL who underwent percutaneous ablation; some patients had multiple ablations (total, 38 ablation procedures), all under general anesthesia. Figure 1 summarizes our case series, number of patients, and number of ablation episodes per patient. Mean (±SD) patient age at the time of first ablation was 39.6 (±18.2) years (Table 1). In general, these relatively young patients did not have major cardiovascular or other major comorbidities, and their preoperative pressures and heart rates with therapy were within the normal range. Primary tumors, the source of metastatic lesions, were most often located in the adrenal glands (29%) or abdomen (38%). For the 21 patients, a total of 38 procedures were performed to treat metastatic lesions. Table 2 summarizes characteristics of metastatic lesions and preoperative medications used to prevent adrenergic response.

**Table 1:** Demographics, preoperative comorbid conditions, hemodynamic parameters, and primary tumor location^a^

Characteristic	At the Time of First Ablation (*n* = 21)
Age, y	39.6 (±18.2)
Male sex	7 (33)
Body mass index, kg/m^2^	26.7 (±6.3)
ASA physical status	
II	13 (62)
III	7 (33)
IV	1 (5)
Coronary artery disease	1 (5)
Anemia^b^	0 (0)
Renal dysfunction^c^	0 (0)
Endocrine disorders	
Hyperparathyroid	3 (14)
Hyperthyroid	1 (5)
Deep vein thrombosis	2 (10)
Lupus erythematosus	1 (5)
Syndromic PPGL (neurofibromatosis type 1)	1 (5)
Preparative hemodynamics	
Blood pressure, mm Hg	
Systolic	110 (±14)
Diastolic	65 (±10)
Mean arterial	79 (±10)
Heart rate, bpm	78 (±12)
Primary tumor location	
Abdomen	8 (38)
Retroperitoneal	3 (14)
Liver	1 (5)
Adrenal	6 (29)
Head/neck	2 (10)
Spine	1 (5)

*Abbreviations*: *ASA* American Society of Anesthesiologists, *PPGL* pheochromocytoma and paraganglioma

^a^Values are mean (±SD) or No. (%)

^b^Anemia defined by hemoglobin levels ≤12 mg/dL for females and ≤13 mg/dL for males

^c^Preoperative kidney function impairment defined as preoperative creatinine concentrations ≥1.4 mg/dL

**Table 2:** Characteristics of metastatic lesions and preoperative medication used to prevent adrenergic response^a^

Characteristic	First Ablation (*n* = 21)	All Ablations (*N* = 38)
Primary tumor location		
Abdomen	8 (38)	15 (39)
Retroperitoneal	3 (14)	6 (16)
Liver	1 (5)	1 (3)
Adrenal	6 (29)	11 (29)
Head/neck	2 (10)	4 (11)
Spine	1 (5)	1 (3)
Procedure		
Cryoablation	5 (24)	10 (26)
RFA	15 (71)	24 (63)
Cryoablation + RFA	0 (0)	4 (11)
Ablation single location		
Vertebral body	3 (14)	8 (21)
Bone	6 (29)	7 (18)
Bladder/urethra	0 (0)	1 (3)
Liver	9 (43)	12 (32)
Ablation multiple locations		
Vertebral body + other bones	1 (5)	8 (21)
Liver + other bones	1 (5)	1 (3)
Adrenal gland + liver	1 (5)	1 (3)
Functional tumors	15 (71)	24 (63)
Preoperative adrenergic blockers		
Any treatment	19 (90)	31 (82)
α-blocker, β-blocker, CCB combinations		
α_1,2_ + β + CCB	0 (0)	2 (5)
α_1_ only	2 (10)	2 (5)
α_1,2_ + β	9 (43)	16 (42)
α_1_ + β	0 (0)	3 (8)
α_1_ + CCB	2 (10)	2 (5)
α_1,2_ only	5 (24)	5 (13)
None (α/β/CCB/metyrosine)	3 (14)	8 (21)
Metyrosine	7 (33)	11 (29)

*Abbreviations*: *CCB* calcium channel blocker, *RFA* radiofrequency ablation

^a^Values are mean (±SD) or No. (%)

Of the 38 ablations performed, 24 had functional metastatic lesions. The catecholamine and metanephrine levels for these patients are shown in Table 3. All patients with functional metastatic tumors had a functional primary PPGL tumor, and in those with multiple metastatic lesions, all were functional. Four patients had nonfunctional primary tumors (2 abdominal and 2 neck PPGLs), and all of their metastatic lesions (in vertebral bodies and ribs) were nonfunctional. In 2 other patients, the primary abdominal PPGL was functional, but the metastatic lesions were nonfunctional (1 with multiple liver metastases, 1 with a sternal metastatic lesion).

**Table 3:** Plasma and urine catecholamine and metanephrine levels in patients with functional metastatic PPGLs^a^

Catecholamine or Metanephrine	Concentration		
	*n*	Median	(Interquartile Range)
Plasma			
Free metanephrine, nmol/L	21	0.2	(0.2–0.3)
Free normetanephrine, nmol/L	21	1.7	(1.1–3.9)
24-Hour urine			
Norepinephrine, mcg/24 h	23	115	(73–165)
Epinephrine, mcg/24 h	23	4.3	(2.6–6.4)
Dopamine, mcg/24 h	23	185	(137–269)
Metanephrine, nmol/L	24	115	(73–164)
Normetanephrine, nmol/L	23	848	(588–1513)
Total metanephrine, mcg/24 h	23	1021	(708–1790)

*Abbreviation*: *PPGLs* pheochromocytomas and paragangliomas

^a^Functional tumors were defined as at least 1 increased preoperative catecholamine or metanephrine level above the reference range

All patients with functional metastatic lesions or with nonfunctional metastases but with a history of a functional PPGL primary tumor received preoperative adrenergic blockade. Among the 4 patients with nonfunctional primary tumors and metastatic lesions, 3 received no pretreatment and 1 received doxazosin prophylactically. Metyrosine was only given to patients with functional tumors: 7 (33%) of first ablations and 11 (29%) of all ablations (Table 2).

### Intraprocedural hemodynamics

**Fig. 2:**
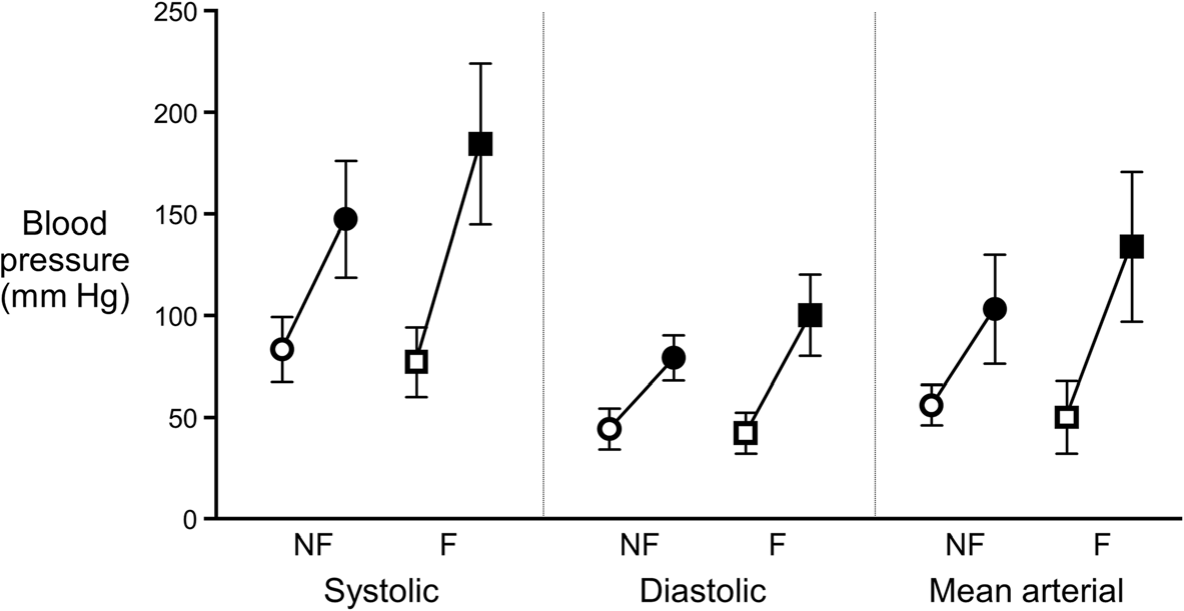
Blood pressure variability. Systolic, diastolic, and mean arterial blood pressure variability during ablation of nonfunctional (NF, circles, *n* = 14) and functional (F, squares, *n* = 24) pheochromocytoma and paraganglioma metastases. Values represent the group mean (±SD) minimum and maximum blood pressure in each category. Open symbols indicate minimum value; closed symbols, maximum value

Intraprocedural variables, type of invasive monitoring, vasoactive treatments, and hemodynamics were compared between patients with functional and nonfunctional metastases (Table 4). The use of potent vasodilators to treat hypertension was more frequent for patients with functional tumors, whereas the use of vasopressors to treat hypotension was comparable between the 2 groups. The values for intraprocedural hemodynamics—maximum systolic, diastolic, and mean arterial blood pressures—also were higher in patients with functional tumors (Table 4). The intraprocedural blood pressure range (within patient difference between maximum and minimum values recorded during the procedure) was greater in patients with functional tumors (Fig. 2, Table 4).

**Table 4:** Intraoperative data and hospital disposition by lesion functional status

Characteristic	First Ablation Episode^a^ (*n* = 21)		*P* value^b^	All Ablation Episodes^a^ (*N* = 38)		*P* value^b^
	Functional Tumor			Functional Tumor		
	No (*n* = 6)	Yes (*n* = 15)		No (*n* = 14)	Yes (*n* = 24)	
Arterial line	3 (50)	14 (93)	0.053	9 (64)	21 (88)	0.12
Central venous catheter	1 (17)	1 (7)	0.50	3 (21)	3 (13)	0.65
Vasodilator, any	2 (33)	10 (67)	0.33	3 (21)	16 (67)	0.02
Nitroprusside	2 (33)	7 (47)		3 (21)	12 (50)	
β-adrenergic blocker	1 (17)	7 (47)		1 (7)	10 (42)	
Clevidipine	0 (0)	1 (7)		1 (7)	1 (4)	
Vasopressor, any	2 (33)	8 (53)	0.64	7 (50)	14 (58)	0.74
Phenylephrine	2 (33)	8 (53)		7 (50)	14 (58)	
Epinephrine	0 (0)	0 (0)		0 (0)	1 (4)	
Vasopressin	0 (0)	0 (0)		0 (0)	1 (4)	
Blood pressure, mm Hg						
Maximum						
Systolic	149 (±36)	176 (±36)	0.15	147 (±29)	184 (±40)	0.005
Diastolic	80 (±12)	102 (±21)	0.02	79 (±11)	100 (±20)	0.001
Mean arterial	98 (±16)	126 (±25)	0.02	103 (±27)	134 (±37)	0.01
Minimum						
Systolic	84 (±11)	79 (±17)	0.49	83 (±16)	77 (±17)	0.31
Diastolic	44 (±10)	44 (±12)	0.94	44 (±10)	42 (±10)	0.69
Mean arterial	57 (±12)	51 (±20)	0.55	56 (±10)	50 (±18)	0.22
Range^c^						
Systolic	65 (±42)	97 (±46)	0.16	64 (±30)	106 (±48)	0.005
Diastolic	36 (±18)	58 (±25)	0.06	35 (±14)	58 (±22)	0.002
Mean arterial	42 (±25)	74 (±35)	0.051	47 (±29)	84 (±43)	0.007
Systolic pressure ≥200 mm Hg	1 (17)	2 (13)	>0.99	1 (7)	7 (29)	0.22
Mean arterial pressure ≤55 mm Hg	4 (67)	8 (53)	0.66	8 (57)	14 (58)	>0.99
Heart rate, beats/min						
Maximum	95 (±28)	96 (±25)	0.91	91 (±22)	95 (±25)	0.62
Minimum	52 (±8)	54 (±11)	0.70	50 (±9)	50 (±10)	0.93
Range	43 (±24)	43 (±28)	0.98	42 (±21)	45 (±27)	0.67
Intraoperative fluid requirement, L	1.6 (±0.9)	1.5 (±0.6)	0.85	1.7 (±0.7)	1.7 (±0.6)	0.96
Intensive care unit admission	0 (0)	1 (7)	>0.99	1 (7)	4 (17)	0.63
Hospital stay after ablation, days^d^			>0.99			0.80
1	4 (67)	8 (53)		9 (64)	13 (57)	
2	1 (17)	4 (27)		2 (14)	6 (26)	
≥3^d^	1 (17)	3 (20)		3 (21)	4 (17)	

^a^Values are No. (%) or mean (±SD)

^b^Fisher exact test for categorical data; 2-sample *t* test for continuous data

^c^Calculated for each patient as maximum value – minimum value

^d^For hospital length of stay (LOS), data are summarized for 37 ablation episodes (14 nonfunctional, 23 functional) where the patient was discharged alive. Seven patients were hospitalized ≥3 days for the following reasons: 2 patients underwent cementoplasty as a separate procedure (LOS, 3 days each); 1 patient received postoperative anticoagulotherapy because of recurrent deep vein thrombosis (LOS, 4 days); 1 patient had postprocedural bleeding (LOS, 3 days); 3 patients had ablation as an initial part of treatment and had other major surgeries during the same hospital stay (corpectomy/discectomy [LOS, 4 days]; wedge resection of metastatic lesions in the liver [LOS, 5 days]; and repair of a ventral hernia [LOS, 5 days])

**Fig. 3:**
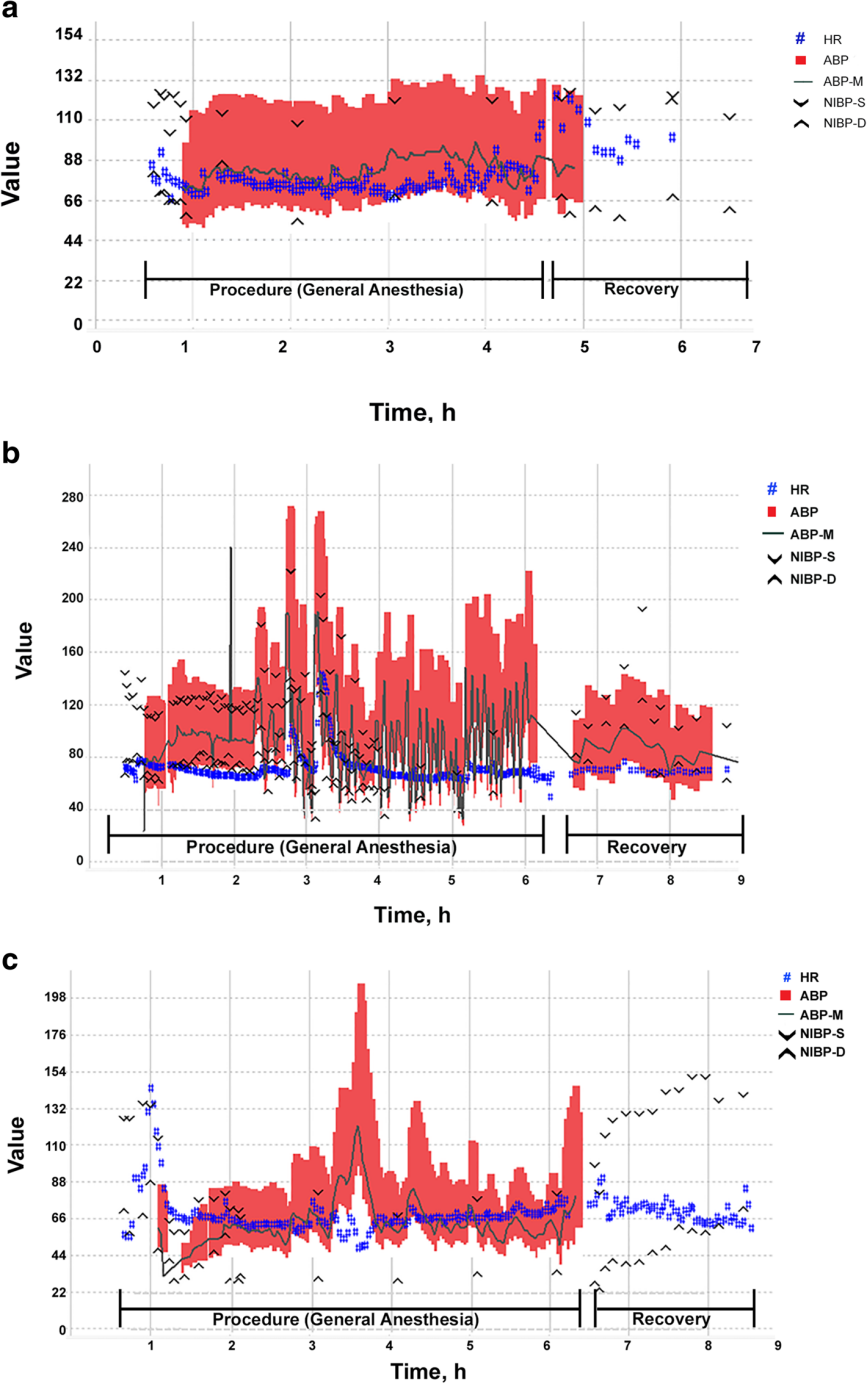
Intraoperative hemodynamics during thermal ablation of pheochromocytoma and paraganglioma (PPGL) metastatic lesions. **a** Patient with nonfunctional primary PPGLs and nonfunctional vertebral body metastasis. **b** Patient with a previously resected functional abdominal PPGL and with functional metastatic lesions. **c** Patient with primary functional PPGL and nonfunctional metastatic liver lesions. ABP indicates blood pressure tracing from arterial line; ABP-M, mean arterial blood pressure; HR, heart rate; NIBP-D, diastolic noninvasive blood pressure; NIBP-S, systolic noninvasive blood pressure. HR is measured in beats/min; all blood pressures are mm Hg

Figure 3 illustrates the intraprocedural hemodynamic course in 3 patients: a patient with a nonfunctional primary tumor in the right carotid body and a nonfunctional vertebral body metastasis (Fig. 3a), a patient with a prior functional abdominal PPGL and functional vertebral and rib metastases (Fig. 3b), and a patient with a functional primary abdominal PPGL treated surgically 6 years before undergoing ablation of 6 nonfunctional liver metastases (Fig. 3c). The patients with functional metastatic lesions (Fig. 3b) and nonfunctional metastatic lesions from a primary PPGL tumor that was functional (Fig. 3c) showed substantial hemodynamic instability during ablation.

Intravenous fluid replacement during procedures was similar between functional and nonfunctional metastatic lesions, and no patients required an intraprocedural transfusion. Hospital length of stay was typically 1 or 2 days, with 7 of 38 ablations (18%) having a hospital stay of 3 or more days. The indications for prolonged hospitalization were unrelated to ablation-associated complications (Table 4 footnote d).

### Procedural complications

Five patients required ICU admission: 3 were admitted for precautionary monitoring, 1 had recalcitrant hypotension requiring vasopressors, and 1 had a hypertensive crisis and required intensive vasodilator management. In addition, postablation bleeding developed in 1 patient, who received a transfusion on a general ward, while 1 patient died intraoperatively. Table 5 provides details for 4 patients with complications including 2 patients with nonprecautionary ICU admission (hypertension and hypotension, 1 each), 1 with postprocedural bleeding, and 1 patient who died from argon gas embolism.

**Table 5:** Postoperative course in 4 patients with complications

Pt	Site of Metastatic Lesion	Preoperative Blockade	Intraoperative Course	Postoperative Course	Complication Outcome
1	T3, T6, T12, L1, L3, and right pubic symphysis functional PPGL	PBZ 20 mg/d Metoprolol 100 mg/d	Profound hypotension treated with phenylephrine	Transferred to ICU; hypotensive with blood pressures 70/50 mm Hg	Hypotension resolved with 6 L crystalloids and repeated doses of midodrine; LOS, 3 d
2	T11 vertebral body functional PPGL	Metyrosine escalating dose 1 to 4 g/d Metoprolol 75 mg/d PBZ 30 mg/d	Hypertension treated with nitroprusside and hydralazine (4 mg)	Transferred to ICU; hypertensive urgency (blood pressures ≈220/100 mm Hg)	Hypertension treated with esmolol 70 mg, hydralazine 9 mg, and metoprolol 7 mg; LOS, 2 d
3	Lumbar and sacral vertebral lesions; functional PPGL	Metyrosine escalating dose 1 to 4 g/d Metoprolol 50 mg/d PBZ 20 mg/d	Equipment malfunction resulting in argon gas embolism	NA	Intraprocedural death
4	Liver and rib lesions; nonfunctional PPGL	None	Bleeding and chest hematoma	Received 3 U of red blood cells and 6 U of platelets for thrombocytopenia	Bleeding was well controlled; LOS, 3 d

*Abbreviations*: *ICU* intensive care unit, *L* lumbar vertebra, *LOS* hospital length of stay, *NA* not applicable, *PBZ* phenoxybenzamine, *PPGL* pheochromocytoma and paraganglioma, *T* thoracic vertebra

## Discussion

Our main finding is that patients with metastatic PPGLs, even when pretreated with adrenergic blocking agents, may experience substantial intraprocedural hemodynamic instability. A nonfunctional metastatic lesion, as assessed from normal preprocedural plasma catecholamine/metanephrine concentrations, does not guarantee intraprocedural hemodynamic stability, especially if the metastatic lesion originates from a primary tumor that was functional. Although percutaneous ablation of metastatic PPGL may be considered a ‘minimally invasive’ intervention, it may be associated with severe complications.

Hemodynamic volatility is common during open or laparoscopic PPGL resection. The concentration of catecholamines in PPGL tumors may be high [21], and intraoperative catecholamine surge has been observed during surgical adrenalectomy of not only functional PPGLs [22] but also PPGLs with negative biochemistry [9, 23–25]. Healthy adrenal glands contain an abundance of catecholamines, and percutaneous ablation of normal adrenal parenchyma can induce their release. In an animal model, Yamakado et al. [26] reported blood pressure and catecholamine surges during radiofrequency ablation of adrenal glands. Espinosa De Ycaza et al.^4^ reviewed hemodynamics during thermal ablation of metastases from renal or lung tumors to adrenal gland, and found that ablation of lesions in adrenal glands may also be associated with hypertensive urgencies. Similarly, Fintelmann et al. [27] examined the risk of catecholamine release during ablation of nonhormonally active adrenal gland metastases, and reported that the presence of normal adrenal tissue and larger tumor diameter were associated with increased catecholamine surge. In contrast to these studies that examined hemodynamics during ablation of metastases of nonhormonally active tumors to adrenal gland, the present study quantifies hemodynamic oscillations during ablation of *extra-adrenal* metastatic lesions, many of which were hormonally active PPGL metastases.

Although plasma catecholamine concentrations during ablation were not measured in our patients, the pattern of intraprocedural hemodynamics was consistent with catecholamine release from PPGL. The large blood pressure oscillations were encountered during ablation of functional metastatic lesions (see Fig. 3b) and also during ablation of nonfunctional metastatic lesions originating from functioning PPGL tumor (see Fig. 3c). This suggests that when anticipating hemodynamic volatility, it is important to review the patient’s history and to consider the secreting functionality of not only treated metastatic lesions but also the originating tumor. Therefore, nonfunctional status of a metastatic lesion, as assessed from nonelevated preprocedural catecholamine/metanephrine levels, cannot guarantee intraprocedural hemodynamic stability [23].

The majority of our patients were pretreated with α-adrenergic blockade, but despite that we still observed large hemodynamic oscillations during ablations. This is consistent with previous observations that preoperative adrenergic blockade cannot entirely eliminate intraoperative hemodynamic oscillations in these patients [13, 15, 16]. In our series, 1 patient was admitted to the ICU after ablation for treatment of severe hypertension despite the fact that he received comprehensive preoperative adrenergic blockade (Table 5). In another patient, systolic blood pressure peaked at 280 mm Hg during ablation despite preprocedural adrenergic blockade. This illustrates that despite preoperative pharmacologic blockade, anesthesiologists must be vigilant and prepared for management of extreme hemodynamic variations when dealing with PPGL metastatic lesions. Furthermore, the maximum blood pressures observed during ablation of functional metastatic lesions were comparable to the values we recently reported for surgical resection of primary functional tumors [16]. This should alert anesthesiologists that when providing anesthesia for ablation of metastatic PPGLs in radiology suites, the preparation for hemodynamic management should match standards used for the surgical resection. However, despite large hemodynamic oscillations during ablations no major cardiovascular complications were noted in our case series, likely because the patients were relatively young and without major preexisting cardiovascular comorbidities.

### Limitations and strengths

The main limitation of our study is related to the historical cohort study design and retrospective retrieval of data. Our study cannot assess the efficacy of preprocedural adrenergic blockade, because the majority of our patients with metastatic PPGL receive these medications. Furthermore, we did not measure intraprocedural serum catecholamines; therefore, we cannot establish causality between observed hemodynamic instability and catecholamine release, although this remains the most plausible explanation, as previously shown [26].

## Conclusion

Patients with functional PPGL metastatic lesions had greater intraprocedural blood pressure variability than those with nonfunctional lesions. A normal preprocedural plasma catecholamine/metanephrine level does not ensure intraprocedural hemodynamic stability, especially if the primary tumor was functional. Although ‘minimally invasive’, percutaneous ablation of metastatic PPGL may be associated with severe complications.

## Abbreviations

ICU: Intensive care unit
PPGLs: Pheochromocytomas and paragangliomas
